# Checklist of the bees (Hymenoptera, Apoidea) of New Caledonia

**DOI:** 10.3897/BDJ.11.e105291

**Published:** 2023-07-31

**Authors:** Marie Zakardjian, Hervé Jourdan, Thomas Cochenille, Prisca Mahé, Benoît Geslin

**Affiliations:** 1 Aix Marseille Univ, Avignon Univ, CNRS, IRD, IMBE, Marseille, France Aix Marseille Univ, Avignon Univ, CNRS, IRD, IMBE Marseille France; 2 Aix Marseille Univ, Avignon Univ, CNRS, IRD, IMBE, Nouméa, France Aix Marseille Univ, Avignon Univ, CNRS, IRD, IMBE Nouméa France

**Keywords:** bee distribution, occurrences, alien species, island ecosystem

## Abstract

**Background:**

In a world where insects and notably bees are declining, assessing their distribution over time and space is crucial to evaluate species status and highlight conservation priorities. However, this can be a daunting task, especially in areas such as tropical oceanic islands where exhaustive samplings over time have been lacking. This is the case in New Caledonia, an archipelago located in the southwest Pacific. Historical records of bee species are piecemeal and, although contemporary samplings have significantly advanced our knowledge of the bee fauna of New Caledonia, the status of several species remains to be elucidated.

**New information:**

Here, we provide an updated checklist of the 51 bee species recorded for New Caledonia using previous publications and personal samplings. We documented their distribution, origin (i.e. endemic, native or alien) and the year and location of their occurrences. Based on the year of their first capture and the year of their last capture, we determined an occurrence status for each species. Thus, 10 years after the last checklist of the New Caledonian bee fauna, the literature review and recent samplings allowed us to add six new species to the list. Half of them are recently introduced species including one firstly mentioned in this paper (i.e. *Hylaeusalbonitens*). We consider here that 30 species are effectively present on the territory and the presence of 21 species could not be determined due to a lack of data, which highlights the need to increase sampling efforts across New Caledonia. Given the difficulty of exhaustively sampling the entire archipelago, we would recommend taking, as a starting point, altitude environments and areas where data-deficient species were captured. In a broader perspective, biomolecular analyses are crucial to confirm species identifications. This is also needed to make comparisons between archipelagoes and thus clarify the distribution and status of species at the scale of the southwest Pacific.

## Introduction

Insects and notably bees are declining worldwide (Wagner 2020, Zattara and Aizen 2021). For 50 years, many studies have reported a decrease in their abundance and species richness, even in protected areas (Hallmann et al. 2017, Seibold et al. 2019). The magnitude and rate of this biodiversity loss have urged taxonomists and naturalists around the globe to exhaustively assess species diversity and their distribution over time and space (Orr et al. 2021). This baseline information is crucial to evaluate species status and provide informative tools for conservation, such as IUCN Red Lists (Rodrigues et al. 2006, Potts et al. 2010, Cardoso et al. 2011, Fox et al. 2019). While in developed countries, where bee species have been studied for a long time, the status of bee populations is already difficult to assess - as a single example, the European Red List of Bees did not evaluate the status of more than a half of the 2000 species present on the continent due to a lack of data (Nieto et al. 2014) - this issue is all the more relevant in other parts of the world where bee species have been seldomly sampled, notably in oceanic tropical and subtropical islands, such as New Caledonia (Orr et al. 2021).

New Caledonia is located in the southwest Pacific. This French overseas archipelago is part of the Australasian biogeographic realm. Historically, the archipelago went through a total submersion event between 75 and 60 Mya and likely at least a partial submersion event between 34 and 25 Mya (Maurizot and Campbell 2020) and has been isolated from any continent since, allowing an important species radiation (Grandcolas et al. 2008, Nattier et al. 2017). The archipelago covers 18 519 km² with a mosaic of edaphic and topographic conditions supporting a high diversity of habitats. Notably, a third of the main island is covered by ultramafic soils, originating from the overlap of peridotites on the continental crust during an obduction event in the late Eocene (Pelletier 2006, Pillon et al. 2020). These soils are characterised by a low nutrient availability and high concentrations of metals and metalloids, strongly constraining plant growth. As such, plants growing on ultramafic substrates present a very high rate of endemism. Thus, New Caledonia is considered as one of the main biodiversity hotspots at global scale for vascular plants with an endemic rate of 74%, up to 96.7% in ultramafic habitats (Morat et al. 2012, Isnard et al. 2016, Munzinger et al. 2022). However, regarding animals, while some groups also display a remarkable diversity and a high rate of endemism (e.g. Squamata; Bernstein et al. (2021), Slavenko et al. (2023)), others, such as bees, appear remarkably poor, illustrating a convincing example of island disharmony (Vargas 2012).

From the end of the 19^th^ century to the middle of the 20^th^ century, very few mentions and descriptions of bees were published for New Caledonia (Vachal 1897, Friese 1905, Vachal 1907, Vachal 1908, Cockerell 1911, von Schulthess 1915, Turner 1919, Cockerell 1929, Cockerell 1939, Cheesman 1953, Donovan 1983). These records are fragmentary and mostly based on the examination of old collections. The first review of the bee species of New Caledonia is attributed to Michener in 1965, who listed nine species (previously mentioned in the literature) and one new species retrieved from an unexamined collection (Michener 1965). Following this, Donovan (1983) made additional captures between 1979 and 1981 and updated the number of New Caledonian bee species to 28, unfortunately naming only two of them. This number did not change until 2003, when Pauly & Muzinger updated this list to 22 species, based on specimens stored in collections and additional captures realised between 1998 and 1999 (Pauly and Munzinger 2003). An important step in the knowledge of the New Caledonian bee fauna was achieved by Donovan et al. (2013), who studied plant-bee interactions using previous collections and additional captures realised between 1979 and 2008. This substantial work resulted in a two-fold increase in the number of bee species recorded for New Caledonia. However, amongst the 43 bee species listed, 24 were undetermined species at this stage. Subsequently, the works of Pauly et al. (Pauly et al. 2013a, Pauly et al. 2013b, Pauly et al. 2015) on the description and revision of New Caledonian *Austronomia*, *Lasioglossum* and *Homalictus* genera allowed a species name to be given to 15 of them. Since then, no review has provided an updated assessment of the bees of New Caledonia.

For the last ten years, we have realised three bee sampling campaigns and opportunistic captures in several localities through New Caledonia (e.g. Zakardjian et al. (2020), Zakardjian et al. (2023a)) and we felt the lack of an updated checklist clarifying the advances of the last 50 years. For example, many bee species recorded for New Caledonia during the 20^th^ century have not been seen for several decades, if not since their first detection. Conversely, new alien bee species did enter the territory (see below). This is why, in this paper, we provide an updated checklist of the bee fauna of New Caledonia. We provide their status, distribution and main synonymies. We also provide every occurrence (date and location) found in the literature and in our samplings, together with a comprehensive list of the plant species they visit.

## Materials and methods

### Literature search

The checklist provided here results from: i) a review of existing literature and ii) additional data from recent field samplings.

Reviewed publications were retrieved from three methods. First, we retrieved 15 publications from GoogleScholar using the key words “New Caledonia” and “bees”. Then, eight were retrieved from the personal library of H. Jourdan. Finally, we retrieved 14 additional publications of interest (i.e. mentioning New Caledonian bee species) cited in the former publications. In total, we scanned 37 publications. Amongst them, 23 were of interest and used in this work (Suppl. material 1). In those publications, we looked for mentions of bee species in New Caledonia. For each bee species, we gathered information concerning their taxonomy (i.e. genus, subgenus, species, subspecies, synonymies and past combinations), occurrences in New Caledonia (i.e. localities and dates of capture), plant species visited, distribution outside New Caledonia if not endemic and original status (i.e. endemic, native, alien). In order to increase the reliability of this information, we verified the valid name and synonymies of each species according to the INPN's (French National Inventory of the Natural Patrimony, https://inpn.mnhn.fr) taxonomic repository TaxRef (Gargominy et al. 2022). For example, one species was named *Lasioglossuminstabilis* (Cockerell 1914) in the publications reviewed (Pauly et al. 2013b). However, according to TaxRef, this name is a synonym and its valid name is *Lasioglossuminstabile* (Cockerell, 1914). Therefore, this species appears as *L.instabile* in this checklist. We also systematically verified species distribution outside New Caledonia using GBIF (Global Biodiversity Information Facility, www.gbif.org) and completed the information based on our own expertise (Hervé Jourdan, pers. com.). We noted every country in which each species has been observed.

### Additional samplings

In addition to the occurrences retrieved from the existing literature, we implemented new occurrences and plant species visited from our personal published (Zakardjian et al. 2020) and unpublished datasets and opportunistic samplings - all realised between 2017 and 2023.

The first dataset comes from a sampling conducted in 2017, from March to May, in Ouvéa, Tiga, Koumac and Belep Islands areas. In each area, bees were captured along 24 transects of 50 m placed to represent their diversity of environments (e.g. forest, coastal vegetation, fallow land, shrubland and subsistence agriculture). Each transect was walked for 25 min during which bees interacting with flowers were captured using nets. In addition, in each site, 16 pan traps (4 blue, 4 red, 4 white and 4 yellow) were distributed during 48 h.

The second dataset comes from a sampling conducted in 2019, from February to April, in Nouméa and the Tontouta Valley. Nouméa is the main city of the archipelago, with 99,926 inhabitants for an area of 45.7 km². Within the city, three sites were sampled, separated by at least 3 km. These three sites were: (i) the park of the French National Research Institute for Sustainable Development (Institut de Recherche pour le Développement); (ii) the Parc Zoologique et Forestier Michel Corbasson and (iii) the Tjibaou Cultural Centre. The Tontouta Valley is located 30 km north of Nouméa. Within the Tontouta Valley, three sites were sampled, separated by at least 3 km. In each site, the plant species presenting the most abundant floral resources were sampled. Depending on the number of available flowering species, up to four plant species were observed during each sampling session. For each plant species, bees contacting flowers in a 1 m² quadrat were captured using nets during 10 minutes. In total, each Tontouta Valley site was sampled 10 times and each Nouméa site 11 times.

The third dataset comes from a sampling realised in 2022, from March to June, in 14 sites along an urbanisation gradient running from Nouméa to the Col de Mouirange (Suppl. material 2). In each site, the plant species presenting the most abundant floral resources were sampled. Depending on the number of available flowering species, up to five plant species were observed during each sampling session. For each plant species, bees contacting flowers in a 1 m² quadrat were captured using nets during 10 minutes. Each site was sampled four times.

Records retrieved those additional samplings have bee published through GBIF (Zakardjian et al. 2023b) and are provided within this checklist together with historical data.

### Occurrence status

For each bee species, we determined an occurrence status in New Caledonia, based on the year of first capture and the year of last capture. Species were determined as "present" if individuals were captured in more than one year including at least one during the last 50 years (i.e. later than 1973) or "data deficient" if individuals were captured in only one year.

### Origin status

Information on the distribution of species was used to determine or correct species origin status. Indeed, for most of the species listed here, their status does not appear in the reviewed publications or may not be valid anymore. For example, *Megachilealbomarginata* was mentioned as endemic to New Caledonia in Pauly and Munzinger (2003). However, a subsequent work showed that this species is also present in Fiji (Davies et al. 2013). Thus, *M.albomarginata* appears as native to New Caledonia in the present checklist. Species present elsewhere than in New Caledonia without any mention of an alien origin in the archipelago were categorised as native. Then, native species only present in New Caledonia were categorised as endemic here. Finally, for alien species, we used the definition given by Russell and Blackburn (2017), according to which a species is considered as alien if its presence is due to a geographical displacement through anthropogenic activities (e.g. voluntary importation, accidental transport via commercial trade). Thus, species that naturally colonised New Caledonia were not considered as alien in this checklist. First, species mentioned as alien or likely alien in literature were systematically categorised in the same way in the checklist. Then, species that were recorded for the first time during our additional samplings were considered as alien.

### Doubtful species

Species for which taxonomic validity and/or for which effective presence in New Caledonia could not be acknowledged with a high degree of confidence (i.e. undetermined species and data-deficient species) are indicated by an asterisk in the checklist.

## Checklists

### Apidae

#### Amegilla (Zonamegilla) pulchra

(Smith, 1854)

33767012-8211-5949-A67C-B849DB0D5017

Anthophora holmesi Rayment, 1947;
Anthophora parapulchra Rayment, 1947;
Anthophora perpulchra wallaciella Rayment, 1947;
Anthophora pulchra townleyella Rayment, 1947;
Anthophora pulchra Smith, 1854;
Anthophora salteri Cockerell, 1905;
Anthophora shafferyella Rayment, 1947;
Amegilla holmesi (Rayment, 1947);
Amegilla parapulchra (Rayment, 1947);
Amegilla salteri (Cockerell, 1905);
Amegilla shafferyella (Rayment, 1947);
Amegilla townleyella (Rayment, 1947);
Amegilla wallaciella (Rayment, 1947)

##### Feeds on

Acanthaceae: *Ruelliasimplex* (alien); Apocynaceae: *Neriumoleander* (alien); Convolvulaceae: *Evolvulus* sp. (alien), *Ipomoea* sp. (alien); Lamiaceae: *Plectranthus* sp. (alien); Lythraceae: *Cuphea* sp. (alien); Rubiaceae: *Hameliapatens* (alien), Rutaceae: *Murrayapaniculata* (alien); Solanaceae: *Solanumlycopersicum* (alien), *Solanumtorvum* (alien); Verbenaceae: *Stachytarphetacayennensis* (alien), *Durantaerecta* (alien) (new records).

##### Native status

Alien

##### Distribution

The subgenus Zonamegilla - Popov, 1950 - is mostly present in Australia (reviewed by Leijs et al. (2017)). This species is therefore present in Australia, but also in Fiji and French Polynesia where it is an alien species (Groom et al. 2017, Groutsch et al. 2018).

Historical data in New Caledonia: Nouméa: Anse Vata, IRD Park, 14 Mar 2019, one female; 16 Mar 2019, one male; two Apr 2019, two males; Parc Zoologique et Forestier, 6 Apr 2019, two males; Rivière Salée, 14 Mar 2022, one individual; 16 Mar 2022, two individuals; Tuband, 16 Mar 2022, five individuals; 25 Mar 2022, one individual; Maison Célières, 18 Mar 2022, five individuals; Normandie, 18 Mar 2022, one individual. Païta: Plaine aux cailloux, Haute Karikouié, pépinière Eriaxis, 05 May 2019. Dumbéa: RM 15, Pépinière Botanea, 05 Feb 2020, two individuals. Mont Dore: Vallon Dore, 30 Mar 2022, one individual; 20 Apr. 2022, one individual (Zakardjian et al. 2023b).

##### Notes

First detected in Nouméa in 2016, the species is now established in the city and has started to spread north along the west coast (Hervé Jourdan, pers. com.).

#### Apis mellifera mellifera

L., 1758

333D2C5F-A182-5FC3-9A32-C7B53753D591

##### Feeds on

A large range of endemic, native and alien plants (including invasive ones).

##### Native status

Alien

##### Distribution

Cosmopolitan subspecies. Ubiquitous in New Caledonia, but absent from Belep Islands and Tiga.

##### Notes

Before the arrival of Europeans, there were no social bees or honey bees in New Caledonia. *Apismelliferamellifera* was first introduced in 1848 from France by priests on Lifu Island for the production of wax candles. It subsequently spread to the mainland and the Isle of Pines in 1853, as well as to Maré during the same period (Lamaignere 2001, Hervé Jourdan, pers. com.). To our knowledge, *Apismellifera* is still absent from Belep Islands and Tiga and abundant elsewhere in the archipelago.

#### Apis mellifera ligustica

Spinola, 1806

E392B6EF-B3AC-58FF-9856-122358972058

##### Feeds on

A large range of endemic, native and alien plants (including invasive ones).

##### Native status

Alien

##### Distribution

Cosmopolitan subspecies. Ubiquitous in New Caleodnia, but absent from Belep islands and Tiga.

##### Notes

*Apismelliferaligustica* was introduced periodically by the end of the 19^th^ and the beginning of 20^th^ centuries on the mainland as mentioned by von Schulthess (1915), but most of colonies were brought from Australia and New Zealand between 1985 and 1988 on the mainland, following American foulbrood outbreaks (Thevenon et al. 1989, Lamaignere 2001).

#### Braunsapis puangensis

(Cockerell, 1929)

6891C0BC-927B-5097-AD7D-A3ED465FAA49

Allodape puangensis Cockerell, 1929

##### Feeds on

Anacardiaceae: *Schinusaroeira* (ex. *Schinusterebinthifolius*) (alien); Araliaceae: *Polysciasscutellaria* (alien); Asteraceae: *Sphagneticolatrilobata* (alien); Lamiaceae: *Ocimumbasilicum* (alien), *Plectranthus* sp. (alien); Lythraceae: *Cuphea* sp. (alien); Portulacaceae: *Portulacaumbraticola* (alien); Rubiaceae: *Hameliapatens* (alien); Solanaceae: *Solanumtorvum* (alien); Verbenaceae: *Clerodendrumugandense* (alien), *Durantaerecta* (alien), *Stachytarphetacayennensis* (alien) (new records).

##### Native status

Alien

##### Distribution

Native range: India, Malaysia, Myanmar, Singapore, Thailand. Alien range: Fiji (da Silva et al. 2016), French Polynesia (Groom et al. 2017).

Historical data in New Caledonia: Nouméa: Anse Vata, IRD park, 16 Mar 2019, one female; 19 Mar 2019, one female; 21 Mar 2019, one male; 23 Mar 2019, one individual; 2 Apr 2019, two females; 10 Apr 2019, two females. Rivière Salée, 16 Mar 2022, two individuals; Tuband, 16 Mar 2022, one individual. Musée de la Ville, 18 Mar 2022, two individuals; 24 Mar 2022, one individual; 27 Apr 2022, three individuals. Magenta Tours, 29 Mar 2022, four individuals; 31 Mar 2022, one individual. Dumbéa: RM 15, Pépinière Botanea, 05 Feb 2020. Four females. Mont Dore: Saint Michel, 20 Apr 2022, four individuals; Vallon Dore, 20 Apr 2022, seven individuals; 21 Apr 2022, one individual; La Conception, 23 Mar 2022, one individual (Zakardjian et al. 2023b).

##### Notes

This species was first detected in New Caledonia in 2019 (Zakardjian et al. 2020). This carpenter bee nests in twigs (Groom et al. 2014b) and displays a casteless sociality (da Silva et al. 2016).

#### Ceratina (Neoceratina) dentipes

Friese, 1914

83AB15DF-4F6F-562A-961A-8C9147802AC2

##### Feeds on

Verbenaceae: *Stachytarphetaaustralis* (alien), *S.cayennensis* (alien) (Donovan et al. 2013); Euphorbiaceae: *Euphorbiatannensis* (alien); Goodeniaceae: *Scaevolasericea* (native); Lythraceae: *Cuphea* sp. (alien); Passifloraceae: *Turneraulmifolia* (alien); Portulacaceae: *Portulacaumbraticola* (alien), *Tribulusrepens* (alien) (new records).

##### Native status

Alien. This species is known to have been introduced in the southWest Pacific (Groom et al. 2017).

##### Distribution

Native range: Hong Kong, Indonesia, Malaysia, Taïwan, Thailand. Alien range: Hawaii, Cook islands, French Polynesia, Mauritius, Vanuatu, Solomon, Fiji, Samoa (Groom et al. 2017, Shell and Rehan 2019).

Historical data in New Caledonia: 16 km SE La Foa, 20-22 Dec 1991, one female. Dec 1979-Sep 2008, three females (Donovan et al. 2013). Tiga, 9 Apr 2017, four females. Koumac, 1 May 2017, one male and one female. Nouméa: Musée de la Ville, 24 Mar 2022, one individual; 27 Apr 2022, one individual; Magenta Tours, 22 Apr 2022, one individual (Zakardjian et al. 2023b).

##### Notes

This carpenter bee nests in twigs. Several individuals may be observed in the same tunnel (i.e. pseudo-social behaviour, see Rehan et al. (2009)).

### Colletidae

#### Euhesma sp.*

9EB29FE6-EDD8-5858-90F5-2D35FBD0349C

##### Feeds on

Cunoniaceae: *Codiaalbifrons* (native); Dilleniaceae: *Hibbertiaheterotricha* (native), *Hibbertiapulchella* (native), *Hibertia* sp. (native); Myrtaceae: *Syzygiumquadrangulare* (native) (Donovan et al. 2013).

##### Distribution

Historical data in New Caledonia: Mont Koghi, 4-6 Oct 1967, one male. Dec 1979-Sep 2008, five females, no location (Donovan et al. 2013).

##### Notes

This species is named *Euhesma* sp. indet. One in Donovan et al. (2013).

No precise location in Donovan et al. (2013). However, the list of plant species described in the latter and the presence of a male in the Mont Koghi suggests that the species can be observed in forested or maquis areas on ultramafic substrates.

#### Euryglossina sp. 1*

AB222A96-D4BD-5081-B035-D5D43A021834

##### Feeds on

Sapindaceae: *Litchichinensis* (alien) (Donovan et al. 2013).

##### Distribution

Historical data in New Caledonia: 3 Jul 1980, one male and one female. Dec 1979-Sep 2008, one male and one female, no location (Donovan et al. 2013).

##### Notes

This species is named *Euryglossina* sp. indet. One in Donovan et al. (2013).

#### Euryglossina sp. 2*

45313935-CA31-56DB-9672-DD4387D39E0E

##### Distribution

Historical data in New Caledonia: Thio: Forêt de Sailles, 9 Dec 2001, two females (Donovan et al. 2013).

##### Notes

This species is named *Euryglossina* sp. indet. Two in Donovan et al. (2013).

**Occurrence status**: data deficient.

#### Hylaeus (Gnathoprosopis) albonitens

(Cockerell, 1905)*

066A2B2A-384F-575E-BDF2-7C802C07B7A0

Prosopis albonitens Cockerell, 1905;
Prosopis albipes , 1924

##### Feeds on

Myrtaceae: *Callistemon* sp. (alien) (new record).

##### Native status

Alien

##### Distribution

Native range: Australia.

Alien range: Hawaii.

Historical data in New Caledonia: Voh, near the cemetary, 8 Dec 2022, one male and three females (Zakardjian et al. 2023b).

##### Notes

Likely a new alien species, potentially introduced via the Vavouto industrial harbour facility. We nevertheless cannot exclude that one of the three below-mentioned *Hylaeus* morphospecies (Donovan et al. 2013) may also fit with *H.albonitens*.

**Occurrence status**: data deficient.

#### Hylaeus sp. 1*

85DBAA79-50AF-505D-82A7-91F478193832

##### Distribution

Historical data in New Caledonia: Ouen Toro, 15 Jan 1972, three females (Donovan et al. 2013).

##### Notes

This species is named *Hylaeus* sp. indet. One in Donovan et al. (2013).

**Occurrence status**: data deficient.

#### Hylaeus sp. 2

*

3B085567-0D43-54AB-9B35-C4C8047F7C50

##### Distribution

Historical data in New Caledonia: Port Laguerre, 11 Apr 2001, one female (Donovan et al. 2013).

##### Notes

This species is named *Hylaeus* sp. indet. Two in Donovan et al. (2013).

**Occurrence status**: data deficient.

#### Hylaeus sp. 3*

43C5E9A4-B922-5458-A6AB-17914D71896E

##### Distribution

Historical data in New Caledonia: Thio: Forêt de Sailles, 3 Dec 2001, one female (Donovan et al. 2013).

##### Notes

This species is named *Hylaeus* sp. indet. Three in Donovan et al. (2013).

**Occurrence status**: data deficient.

#### Leioproctus (Lamprocolletes) pacificus

Michener, 1965

C33B2348-9BEE-5700-B85F-153CD6B6B754

##### Feeds on

Araliaceae: *Myodocarpusfraxinifolius* (native); Celastraceae: *Peripterygiamarginata* (native) (Donovan et al. 2013).

##### Native status

Endemic

##### Distribution

Historical data in New Caledonia: Nepoui Valley, Jul 1940, one male (Pauly and Munzinger 2003, Donovan et al. 2013). Dec 1979-Sep 2008, one male and one female, no location (Donovan et al. 2013).

##### Notes

This species has been recorded in forested or maquis areas including in altitudes (up to 900 m). The recorded host plants and known localities are restricted to ultramafic substrates.

#### Leioproctus (Lamprocolletes) sp.*

7410FDBC-E7BD-5249-8827-49319DB716C8

##### Notes

This species is named *Leioproctus* sp. indet. Two in Donovan et al. (2013).

**Occurrences**: Mont Khogis, 1 Nov 1992, one male (Donovan et al. 2013).

**Occurrence status**: data deficient.

#### Palaeorhiza (Palaeorhiza) flavomellea

Cockerell, 1910

44722344-007A-5E9D-AC75-286BF3EDC6B4

##### Feeds on

Anacardiaceae: *Mangiferaindica* (alien), *Schinusaoeira* (alien); Arecaceae: *Cocosnucifera* (alien); Combretaceae: *Terminalia catappa* (alien); Elaeocarpaceae: *Elaeocarpusangustifolius* (native); Hernandiceae: *Hernandianymphaeifolia* (native), *Hernandiaovigera* (native) (Donovan et al. 2013); Hernandiaceae: *Hernandiaguianensis* (alien) (Pauly and Munzinger 2003); Anacardiceae: *Schinusaroeira* (ex. *Schinusterebinthifolius*) (alien); Araliaceae: *Polysciasscutellaria* (alien); Rubiaceae: *Hameliapatens* (alien) (new records).

##### Native status

Likely alien. Donovan (1983) hypothesised that this species could be alien because individuals were captured in a few localities in the vincinity of Noumea, suggesting a recent establishment.

##### Distribution

Australia.

Historical data in New Caledonia: Nouméa, 27 May 1977, three males and two females (Pauly and Munzinger 2003, Donovan et al. 2013). Nouméa, 17 Feb 1999, five males (Pauly and Munzinger 2003). Dec 1979-Sep 2008, 14 males and two females, no location (Donovan et al. 2013). Ouvéa: Hwadrilla, Hotel Beaupré, 9 Mar 2017, two males and one female. Mont Dore: Rue André Burck, 13 Apr 2022, two individuals. Nouméa: Tuband, 19 Apr 2022, three individuals; Musée de la Ville, 27 Apr 2022, one individual (Zakardjian et al. 2023b).

##### Notes

The alien status remains to be confirmed.

### Halictidae

#### Austronomia cheesmanae

(Michener, 1965)

C2FD8619-1847-5B81-B6AC-72F70C95476E

Lipotriches cheesmanae (Michener, 1965);
Nomia cheesmanae Michener, 1965;
Nomia nuda Cheesman, 1953

##### Feeds on

Myoporaceae: *Myoporumcrassifolium* (native) (Donovan et al. 2013); Arecaceae: *Cocosnucifera* (alien) (new record).

##### Native status

Endemic

##### Distribution

Historical data in New Caledonia: Lifou, Cap des Pins, 18 Nov 1949-18 jan. 1950, one female. Lifou, We, 30-31 Jan 1962, one female; Feb 1962, two males; 16-18 Feb 1963, two males and 3 females. Ouvea, Fayaoué, Jan 1969, two males. Maré, 13 Nov 2002, one female. Lifou, 2006, four females. Lifou, Koumo, 7 Dec 2006, two males and two females (Pauly et al. 2013a). Lifou, 18 Nov 1950, one female. Dec 1979-Sep 2008, one female (Donovan et al. 2013). Ouvéa: Hwadrila 7 Mar. 2017, one female (Zakardjian et al. 2023b).

#### Austronomia doensis

Donovan, Pauly & Munzinger, 2013

3EB95212-A459-555D-B968-AFD263E58372

##### Feeds on

*Sennaoccidentalis* (alien) (Donovan et al. 2013); *Senna* sp. (Pauly et al. 2013a); Polygonaceae: *Antigonumleptotus* (alien) (new records).

##### Native status

Endemic

##### Distribution

Historical data in New Caledonia: Dec 1979-Sep 2008, one female (Donovan et al. 2013). Mt Do, 15 Dec 2000, one female (holotype). Sarraméa, forêt du col d’Amieu, 30-31 Dec 2005, four males and one female (paratype; Pauly et al. 2013a). Koumac, 1 May 2017, one female (Zakardjian et al. 2023b).

#### Austronomia loyali

Donovan, Pauly & Munzinger, 2013

40B5CBF9-F35F-5138-BC00-7BBCF3076527

##### Native status

Native

##### Distribution

Vanuatu

Historical data in New Caledonia: Lifou, Cap des Pins, 18 Nov 1949-18 Jan 1950, two males. Lifou, We, 30-31 Jan 1962, 32 females; Feb 1962, two males and five females; 16-18 Feb 1963, two females; 1 Aug 2003, two females. Ouvea, Fayaoue, Feb 1963, one female. Lifou, 26-27 Mar 1968, one female (holotype; Pauly et al. (2013a)).

#### Austronomia neocaledonica

Donovan, Pauly & Munzinger, 2013 *

3CA082C8-D60A-52E2-9F1C-2A064AF92E99

##### Native status

Endemic

##### Distribution

Historical data in New Caledonia: Rivière Bleue, 3 Nov 1992, one male and one female (paratypes). Mt Koghis, 17 km NE Nouméa, 22 Dec 1992, one male (holotype; Pauly et al. (2013a)).

##### Notes

**Occurrence status**: data deficient.

#### Austronomia sicheli

(Vachal, 1897)

AF51F60E-F75D-58D2-8F06-5270379007A5

Nomia sicheli Vachal, 1897;
Nomia wilmattae Cockerell, 1929

##### Feeds on

*Polysciaspancheri* (Schlessman et al. 1990); Melastomataceae: *Melastomadenticulatum* (native); Violaceae: *Agatealongipedicellata* (native); *Babingtonia* sp., *Myoporumcrassifolium*, *Poinsettia* sp., *Eugenia* sp., *Scaevola* sp., *Stypheliapancheri* (native) (Pauly & Muzinger 2003); Anacardiaceae: *Schinusterebinthifolia* (alien); Apocynaceae: *Parsonsiacrebriflora* (native); Araliaceae: *Polysciassessiliflora* (native), *Polyscias* sp. (native), unknown (native); Asteraceae: *Ageratumconyzoides* (alien); Celastraceae: *Peripterygiamarginata* (native); Combretaceae: *Lumnitzeraracemosa* (native); Connaraceae: unknown (native); Cunoniaceae: *Codiadiscolor* (native); Dilleniaceae: *Hibbertiabouletii* (native), *Hibbertialucens* (native), *Hibbertiapancheri* (native), *Hibbertiapulchella* (native), *Hibbertia* sp. (native); Ericaceae: *Dracophyllumverticillatum* (native), Stypheliacf.cymbulae (native); Escalloniaceae: *Argophyllummontanum* (native); NA: *Cassiafistula* (native), *Cassia* sp. (native), *Storckiellapancheri* (native), *Storckiella* sp. (native); Euphorbiaceae: *Euphorbia* sp. (alien); Goodeniaceae: *Scaevolabeckii* (native), *Scaevolacylindrica* (native), *Scaevolaerosa* (native), *Scaevolamontana* (native); Joinvilleaceae: *Joinvillea* sp. (native); Lamiaceae: *Mentha* sp. (alien); Loganiaceae: *Geniostomadensiflorum* (native); Malpighiaceae: *Tristellateiaaustralasiae* (native); Melastomataceae: *Melastomamalabathricum* (native); Mimosaceae: *Acaciaspirorbis* (native); *Leucaenaleucocephala* (alien), *Mimosadiplotricha* (alien); Myodocarpaceae: *Myodocarpusfraxinifolius* (native); Myrsinaceae: *Tapeinospermaoblongifolium* (native); Myrtaceae: *Cloeziaartensis* (native), *Cloezia* sp. (native), *Melaleucagnidioides* (native), *Melaleucaquinquenervia* (native), *Myrtastrumrufopunctatum* (native), *Myrtus* sp. (native), *Psidiumguajava* (alien), Sannanthaleratii (native), *Sannantha* sp. (native), *Syzygiumjambolanum* (alien), *Syzygiumlateriflorum* (native), *Syzygiumquadrangulare* (native), *Tristaniopsiscalobuxus* (native), *Tristaniopsisglauca* (native), *Tristaniopsisvieillardii* (native), *Uromyrtusemarginata* (native), unknown 1 (native), unknown 2 (native); Phrymaceae: *Mimulus* sp. (alien); Proteaceae: *Grevilleaexul* (native), *Stenocarpusmilnei* (native), *Stenocarpusumbelliferus* (native), *Stenocarpus* sp. (native); Rhamnaceae: *Alphitonianeocaledonica* (native); Rubiaceae: *Normandianeocaledonica* (native); Sapindaceae: *Guioavillosa* (native), *Litchichinensis* (alien), *Storthocalyxpancheri* (native); *Sennaoccidentalis* (alien); Solanaceae: *Solanumtorvum* (alien), unknown (alien); Verbenaceae: *Stachytarphetaindica* (alien); Convolvulaceae: *Ipomoea* sp. (Donovan et al. 2013); *Antigonumleptotus*, *Aryteraarcuata*, Croton, *Euphorbiacyathophora*, *Gymnostomapoissonianum*, *Myrtopsis* sp., *Xanthostemon* sp. (new records).

##### Native status

Endemic

##### Distribution

Historical data in New Caledonia: Nouméa, 23 Jan 1914. Baie Ngo, 10 Feb 1914. Baie Ouemo, 28 Mar 1914 (Turner 1919). Plum Farm, 30 May 1927, one female; 4 Jun 1927, two females. Rivière Bleue, 10 Jan 1999, seven males and 13 females; 11 Jan 1999, three males and 8 females. Mandjelia, 28 Jan 1999, one female. Prony, 10 Feb 1999, two females. Paagoumene, 12 Mar 1999, one male (Pauly and Munzinger 2003). Mt Tinchialit, Sep 1945, three females. Puébo, two females. Cap des Pins, 18 Nov 1950, two males and two females (Cheesman 1953). Near Yaté, 30 Dec 1979, one individual (Donovan 1983). Dec 1979-Sep 2008, 42 males and 147 females (Donovan et al. 2013). Rivière Bleue, Nov-Dec 1985 (Schlessman et al. 1990). Belep: Île Art, 28 Feb 2017, two males and three females; 25 Apr 2017, one male, two females and one individual; 27 Apr 2017, three females; (no date), one male and one female. Ouvéa, 7 Mar 2017, one male; 8 Mar 2017, one individual. Tiga, 8 Apr 2017, four females; 9 Apr 2017, two females; 11 Apr 2017, one male. Koumac, 30 Apr 2017, one male; 1 May 2017, one female. Païta - La Tontouta: Tontouta Valley, 15 Mar 2019, one female; 29 Mar 2019, one female; 1 Apr 2019, one female. Nouméa: Parc Zoologique et Forestier, 30 Mar 2019, one male and two females; 06 Apr 2019, 2 females; Anse Vata, IRD Park, 21 Mar 2019, 1 female. (Zakardjian et al. 2023b).

#### Homalictus aponi

(Cheesman & Perkins, 1939)

A01BB4E6-D992-584D-A16A-9D7802F7B045

##### Feeds on

Amaranthaceae: *Achyranthesaspera* (alien); Anacardiaceae: *Schinusterebinthifolia* (alien); Araliaceae: *Polyscias* sp. (native); Asteraceae: *Bidenspilosa* (alien), *Blumealacera* (native), *Conyza* sp. (alien), *Calendula* sp. (alien), *Emiliasonchifolia* (alien), *Tridaxprocumbens* (alien), *Tridax* sp. (alien), *Youngiajaponica* (alien); Brassicaceae: *Lepidiumvirginicum* (alien); Caesalpiniaceae: *Crotalaria* sp. (alien); Cunoniaceae: *Geissois* sp. (native), *Pancheriabillardierei* (native); Dilleniaceae: *Hibbertiadeplancheana* (native), *Hibbertialucens* (native), *Hibbertiapodocarpifolia* (native), *Hibbertiapulchella* (native), *Hibbertiatontoutensis* (native), *Hibbertia* sp. (native); Ericaceae: Stypheliacf.cymbulae (native); Euphorbiaceae: *Euphorbiahypericifolia* (alien), *Euphorbialophogona* (alien), unknown (native); Goodeniaceae: *Scaevolabeckii* (native), *Scaevolamontana* (native), *Scaevola* sp. (native); Loganiaceae: *Geniostomadensiflora* (native); Malpighiaceae: *Acridocarpusaustrocaledonicus* (native); Malvaceae: *Sidaacuta* (alien), *Sidarhombifolia* (alien); Mimosaceae: *Acaciaspirorbis* (native), *Leucaenaleucocephala* (alien), *Mimosadiplotricha* (alien); Myrtaceae: *Melaleucaquinquenervia* (native), *Metrosiderosoperculata* (native), *Sannanthaleratii* (native), *Sannanthavirgata* (native), *Xanthostemon* sp. (native); Onagraceae: *Bougainvillea* sp. (alien), *Ludwigiaoctovalvis* (native); Orchidaceae: *Eriaxisrigida* (native); Papaveraceae: *Argemonemexicana* (alien); Proteaceae: *Stenocarpusphyllodineus* (native); Rubiaceae: *Normandianeocaledonica* (native); Sapotaceae: *Leptostylispetiolata* (native); Solanaceae: *Solanumlycopersicum* (alien), *Solanumtorvum* (alien), *Solanum* sp. (alien); Tiliaceae: *Corchorus* sp. (native); Verbenaceae: *Durantaerecta* (alien), *Stachytarphetaindica* (alien), *Verbena* sp. (alien); Violaceae: *Agatealongipedicellata* (native); Zygophyllaceae: *Tribuluscistoides* (alien) (Donovan et al. 2013).

##### Native status

Native

##### Distribution

Vanuatu

##### Notes

This bee appears as the most common native bee in New Caledonia. It has been observed in a wide range of habitats (including urban, non-ultramafic, ultramafic and altitude environments) in the whole archipelago.

Potential confusion with *Homalictusurbanus* from Australia; Pauly and Villemant (2009) highlighted a slight difference between the specimens of Vanuatu and New Caledonia compared to specimens from Australia. Based on preliminary biomolecular analysis (unpublished data), we confirm that *H.aponi* is a different species from *H.urbanus* and we confirm its status as a native species from Vanuatu and New Caledonia.

#### Homalictus cocos

Pauly & Munzinger, 2003

BED028E5-BAFC-5500-8EE6-720A83437399

##### Feeds on

Anacardiceae: *Schinusaroeira* (ex. *Schinusterebinthifolius*) (alien); Araliaceae: *Meryta* sp. (native); Arecaceae: *Cocosnucifera*; Schinusaoeira; Euphorbiaceae: *Euphorbia* sp. (alien); Verbenaceae: *Verbena* sp. (alien) (Donovan et al. 2013); Euphorbiaceae: *Euphorbia* sp. (ex. *Poinsettia* sp.) (alien) (Pauly and Munzinger 2003); Araliaceae: *Meryta* sp. (endemic); Malvaceae: *Hibiscustiliaceus* (native) (Pauly et al. 2015); Euphorbiaceae: *Erythrina* sp. (alien) (new record).

##### Native status

Endemic

##### Distribution

Historical data in New Caledonia: Saint Louis, 17 Aug 1940, one female (Donovan et al. 2013, Pauly et al. 2015). Ile des Pins, 23 Oct 1940, three males; 24 Oct 1940, one male and one female; 25 Oct 1940, one male and two females; 26 Oct 1980, 12 males and three females. Nouméa, Feb 1959, one male. Lifou, We, 30-31 Jan 1962, one male and two females. Tao, 8-10 Feb 1963, one male. Anse Vata, 27 Feb 1963, one female; 26 Mar 2006, one female. Ile des Pins, Point SW Kuto, 17 Aug 1979, one male and one female. Bourail, plage de Poé, 3 Jan 2006, six males and five females. Saint Louis, 4 Sep 2007, one female. (Pauly et al. 2015). Nouméa, 23 Apr 1965, one female; 15 Aug 1966, one female; 4 Apr 1969, one female; 27 May 1977, one female (Pauly and Munzinger 2003). Dec 1979-Sep 2008, three males and nine females (Donovan et al. 2013). Nouméa: Centre culturel Tjibaou, 21 Mar 2019, one male. Anse Vata, IRD Park, 27 Mar 2019, one female (Zakardjian et al. 2023b).

#### Homalictus curvistriae

Donovan & Pauly, 2015

0C4316C2-A0AE-519B-8494-2E7DAD2D0438

##### Feeds on

Asteraceae: undetermined species (alien); Cunoniaceae: *Pancheriarobusta* (endemic); Dilleniaceae: *Hibbertialucens* (native), *Hibbertiatrachyphylla* (native); Ericaceae: *Dracophylluminvolucratum* (endemic); Goodeniaceae: *Scaevolabeckii* (endemic); Loganiaceae: *Geniostomadensiflorum* (endemic); Myrtaceae: Metrosiderosoperculata (native), Metrosideros punctata (endemic); Rubiaceae: Normandianeocaledonica (endemic), Psychotria rupicola (endemic); Rutaceae: *Zanthoxyllum* sp. (endemic) (Donovan et al. 2013); Ericaceae: *Dracophyllumverticillatum* (endemic), Stypheliacfcymbulae (endemic); Melastomataceae: *Melastomamalabathricum* (ex. *Melastomadenticulatum*) (native) (Pauly et al. 2015); Goodeniaceae: *Scaevolamontana* (endemic) (new records).

##### Native status

Endemic

##### Distribution

Historical data in New Caledonia: Mt Panié, 8-9 Feb 1963, two males and three females. Rivière Bleue, 14 Nov 1963, one female. Tchambouenne 7 km S, 28 Jan 1964, one female. Mt Koghi, 23-27 Oct 1967, one male. W. of Ponerihouen, 31 Jul 1971, one female. Dumbea, 19 Dec 1979, one female. Montagne des sources, 29 Dec 1979, three males and one female. Mont Dzumac, 11 Oct 1980, one female. Montagne des Sources, 22 Oct 1980, one female. Montagne des Sources, 25 Oct 1980, seven females. Koum Riv., 23 Nov 2001, one female. Haute Vallée de la Ni, 23 Oct 2004, one male and one female. Col de Yaté, 14 Nov 2004, one male. Vallée de la Tchamba, 26 Jul 2005, one male. Prony, 21 Aug 2005, one female. Haute Kuébuni, 25 Mar 2007, one female. Boulinda, 2 Sep 2009, one female. Aoupinié, 3 Sep 2010, one female (Pauly et al. 2015). Dec 1979-Sep. 2008, two males and 12 females (Donovan et al. 2013). Mont Dore, 10 May, 2022, four individuals; 17 May 2022, five individuals; 18 May 2022, two individuals (Zakardjian et al. 2023b).

##### Notes

This species has been observed in forested areas, on ultramafic subtrates, but also volcano sedimentary substrates, mostly in altitudes (> 500 m).

#### Homalictus heliotropiae

Pauly & Donovan, 2015

D6A20CF6-E727-554F-BF92-D72A5A193568

##### Feeds on

Arecaceae: *Cocosnucifera* (alien); Boraginaceae: *Heliotropiumfoertherianum* (ex. *Argusiaargentea*) (native); Myrtaceae: Sannanthaleratii (native) (Donovan et al. 2013).

##### Native status

Endemic

##### Distribution

Historical data in New Caledonia: Dec 1979-Sep 2008, one male and two females. Mou, 25 Dec 1979, three females. Houailou, 25 Dec 1979, one male (Donovan et al. 2013). Yaté, gîte de Port Boisé, 19 Jan 2006, one female (paratype). Presqu’île de Kuébuni, 19 Aug 2007, one female (holotype; Pauly et al. (2015)).

##### Notes

According to known host plant species, this species was observed on south and east coastal habitats on calcareous uplifted reefs.

#### Homalictus hienghenensis

Donovan & Pauly, 2015*

86CB60EB-D4F7-5388-8C1D-3AE2EB003886

##### Native status

Endemic

##### Distribution

Historical data in New Caledonia: Hienghène, 25 Nov 1958, two females (holotype and paratype; Pauly et al. (2015)).

##### Notes

**Occurrence status**: data deficient.

#### Homalictus koghiensis

Donovan & Pauly, 2015*

F5140C8C-3B2A-5E30-A674-BEDDA90AF5E3

##### Native status

Endemic

##### Distribution

Historical data in New Caledonia: Mts Koghis, Jan 1969, three males (holotype and paratypes; Pauly et al. (2015)).

##### Notes

This species was observed in altitudinal (> 500 m) forested areas on ultramafic substrates.

**Occurrence status**: data deficient.

#### Homalictus mcphersoni

Donovan & Pauly, 2015

CCF18768-9A41-5571-82F2-F4CCB74A920A

##### Feeds on

Araliaceae: *Polysciassessiliflora* (endemic), *Scheffleravieillardii* (endemic); Arecaceae: *Cyphokentiacerifera* (endemic); Cunoniaceae: *Cunoniabalansae* (endemic), *Geissoisracemosa* (endemic); Myrtaceae: *Metrosideros* sp. (endemic) (Donovan et al. 2013), Myrtaceae: *Longetiabuxoides* (Pauly et al. 2015).

##### Native status

Endemic

##### Distribution

Historical data in New Caledonia: Mt Koghi, Dec 1963, one female. Yiambi, NE, 14 Oct 1967, one female. Mts des Koghis, Jan 1969, one female. Col des Roussettes, 3 Feb 1971, one female. Mt Dzumac, 24 Feb 1980, three females (including holotype). Mandjelia Forest, 12 Apr 1980, one female. Mt Khogis, 25 Jan 1996, (no number of individuals). Kaala, 8 Dec 2000, six females. Col d’Amos, 16 Nov 2002, one female. Dzumac, 27 Oct 2004, one female. Forêt Nord, 4 Jan 2005, one female. Ponérihouen, forêt de l'Aoupinié, 12-24 Jan 2006, five females. Sarraméa, forêt du col d'Amieu, 14-27 Jan 2006, one female. Plateau de Boakaine, 27 Feb 2013, two females (paratypes; Pauly et al. 2015). Dec 1979-Sep 2008, six females (Donovan et al. 2013).

##### Notes

This species has been observed in forested areas on both volcano-sedimentary and ultramafic substrates, mostly in altitudes (> 500 m).

#### Homalictus melanasiae

Donovan & Pauly, 2015

3AAE6D6E-7FE1-544F-9135-97D090C91133

##### Feeds on

Asteraceae: *Argeratum* sp. (alien); Araliaceae: *Polysciassessiliflora* (endemic), *Polyscias* sp. (endemic); Myodocarpaceae: *Myodocarpus* sp. (endemic); Rhamnaceae: *Alphitonianeocaledonica* (endemic); Sapindaceae: *Guioavillosa* (endemic), *Litchichinensis* (alien) (Donovan et al. 2013).

##### Native status

Endemic

##### Distribution

Historical data in New Caledonia: Thi River Valley, 6 Nov 1940, one female. Mt Koghi, 1 Feb 1961, one female; 8 Oct 1969, four females. Mt Dzumac, 24 Feb 1980, one female. Thy Valley, 18 Jun 1980, one female; 3 Jul 1980, one female; 5 May 1981, five females (including holotype). Col de Mouirange, 10 Oct 1980, one female. Mts Koghis, Auberge, 23 Jul 2003, one female. Houailou, 30 Jul 2003, one female (paratypes). Haute Vallée de la Ni, 29 Apr 2004, one female. Piste Ni-Dzumacs, 7 May 2004, one female (Pauly et al. 2015). Dec 1979-Sep 2008, 11 females (Donovan et al. 2013).

##### Notes

This species has been observed in ultramafic substrates in both maquis and forested areas.

#### Homalictus projectio

Donovan & Pauly, 2015

998254E4-3323-5662-A67D-B581D7AE4C74

##### Native status

Endemic

##### Distribution

Historical data in New Caledonia: Col des Roussettes, 4-6 Feb 1963, one female (holotype). Mt Dzumac, 11 Oct 1980, one female (paratype; Pauly et al. (2015)).

##### Notes

This species has been observed in forested areas on both ultramafic and volcano sedimentary substrates, in high altitudes (450 to 900 m).

#### Homalictus risbeci

(Cockerell, 1929)

BF294F69-A376-5778-B445-E6537C4B187A

Halictus crotalariae Cockerell, 1929;
Halictus risbeci Cockerell, 1929

##### Feeds on

*Crotalaria* sp. (alien) (Cockerell 1929); Araliaceae: *Polysciassessiliflora* (native), *Polyscias* sp. (native), unknown (native); Asteraceae: *Blumealacera* (native); Cunoniaceae: *Geissois* sp. (native), *Pancheriaalaternoides* (native), *Pancheriabillardierei* (native), *Pancheriaphylliraeoides* (native), *Pancheria* sp. (native); Dilleniaceae: *Hibbertialucens* (native), *Hibbertiapancheri* (native), *Hibbertia* sp. (native); Ericaceae: *Dracophyllumverticillatum* (native); *Acaciaspirorbis* (native); Goodeniaceae: *Scaevolabeckii* (native), *Scaevolacylindrica* (native), *Scaevolamontana* (native), *Scaevola* sp. (native); Laxmanniaceae: *Cordyline* sp. (native); Liliaceae: *Rhuacophilajavanica* (native); Linaceae: *Hugoniapenicillanthemum* (native); Malpighiaceae: *Tristellateiaaustralasiae* (native); Malvaceae: *Melochiaodorata* (native); Melastomataceae: *Melastomamalabathricum* (native); Myrtaceae: *Cloeziafloribunda* (native), *Melaleucaquinquenervia* (native), *Metrosiderosoperculata* (native), *Sannanthaleratii* (native), *Syzygium* sp. (native), *Tristaniopsiscalobuxus* (native); Onagraceae: *Ludwigiaoctovalvis* (native); Proteaceae: *Grevillea* sp. (native), *Stenocarpusphyllodineus* (native); Rhizophoraceae: *Rhizophoraapiculata* (native); Rubiceae: *Normandianeocaledonica* (native); Sapindaceae: *Cupaniopsismyrmoctona* (native), *Guioavillosa* (native); Surianaceae: *Surianamaritima* (native); Asteraceae: *Cosmossulphureus* (alien); Brassicaceae: *Brassica* sp. (alien); *Cassiafistula* (alien); *Leucaenaleucocephala* (alien); *Mimosadiplotricha* (alien); Lythraceae: *Lagerstroemiaindica* (alien); Rutaceae: *Citrus* sp. (alien); Verbenaceae: *Stachytarpheta* sp. (alien) (Donovan et al. 2013); *Babingtonialeratii* (Pauly and Munzinger 2003), *Aryteraarcuata*, *Erythrina* sp., *Gymnostomapoissonianum* (new records).

##### Native status

Endemic

##### Notes

This species is widespread in the archipelago and has a broad range of host plants, including endemic, native and alien species. It can be found from low to high altitudes on both ultramafic and volcano sedimentary substrates.

#### Homalictus risbeci crotalariae

(Cockerell, 1929)

3A26E9C6-E3C1-5EBC-AD44-4130ADC65B4E

##### Feeds on

*Alectryoncarinatum*, *Brassica* sp., Eugeniacf.gacognei, *Rhizophoraapiculata*, *Xanthostemon* sp. (Pauly et al. 2015).

##### Native status

Endemic

##### Distribution

Historical data in New Caledonia: Ile de Mouac, 19 Oct 1958, one male. Maré, La Roche, Mar 1959, six males and three females. Lifou, We, 30-31 Jan 1962, three males and one female; Feb 1962, one male and nine females; 16-18 Feb 1963, one female; 1 Aug 2003, one female. Poum, Golone, 29 Jul 2005, one female. Ile Leprédour, 22 Jan 2008, one female; two females. Poum, 3 Apr 2012, one female (Pauly et al. 2015). Belep: Île Art, 28 Feb 2017, seven females; 1 Mar 2017, one female; two Mar 2017, two females; 27 Apr 2017, four females. Ouvea, 16 Mar 2017, one female (Zakardjian et al. 2023b).

##### Notes

The status of *H.risbescicrotalariae* as a subspecies remains to be confirmed through molecular analysis.

#### Homalictus sp. 1*

E996533D-980C-53E7-89D8-B00FA1F30FC8

##### Distribution

Historical data in New Caledonia: Île Mouac, 19 Oct 1958, one male (Donovan et al. 2013)

##### Notes

This species is named *Homalictus* sp. indet. Eleven in Donovan et al. (2013).

**Occurrence status**: data deficient.

#### Homalictus sp. 2*

B1A7EF9B-C867-56BB-AD60-F9C717243239

##### Distribution

Historical data in New Caledonia: Monts des Koghis, Jan 1969, three males (Donovan et al. 2013).

##### Notes

This species is named *Homalictus* sp. indet. Seven in Donovan et al. (2013).

**Occurrence status**: data deficient.

#### Lasioglossum (Austrevylaeus) sp.*

40AC1A25-35FE-50CF-9B6A-4D294ABF49C4

##### Notes

This species is mentioned in Donovan (1983) based on a personal communication. Pauly and Munzinger (2003) stated that they did not examine the species which, therefore, needs confirmation.

**Occurrence status**: data deficient.

#### Lasioglossum (Chilalictus) alticola

Pauly, Walker, Munzinger & Donovan, 2013

03C5CAE4-5D4E-5EF1-82C7-C4F7C775284D

##### Feeds on

Apocynaceae: *Parsonsia* sp. (endemic); Araliaceae: *Polysciasdioica* (endemic); Dilleniaceae: *Hibbertianana* (endemic); Ericaceae: *Dracophylluminvolucratum* (endemic); Liliaceae: *Rhuacophilajavanica* (native); Phellinaceae: *Phellinelucida* (native) (Donovan et al. 2013); Asphodelaceae: *Rhuacophilajavanica* (endemic); Phellinaceae: *Phellinelucida* (endemic); Proteaceae: *Beaupreapancheri* (endemic) (Pauly et al. 2013b). According to pollen analysis, it also visits Arecaceae, Myrsinae, Rubiaceae and Elaeocarpaceae (Pauly et al. 2013b).

##### Native status

Endemic

##### Distribution

Historical data in New Caledonia: Dec 1979-Sep 2008, one male and 11 females (Donovan et al. 2013). Mt Ouin, 2 Dec 2000, one female. Aoupinié, 3-23 Nov 2001, one male and eight females. Forêt de Saille, 9 Dec 2001, one female (holotype). Mt. Humboldt, 5-8 Nov 2002, six females. Mont Kouakoué, 25 Nov-2 Dec 2002, four females. Monts Dzumac, 4 Dec 2002, two females. Mont Mou, 17 Jan 2004, three females. Haute-Ni, 25 Oct 2004, one female. Kouakoué, south face, 13 May 2006, one female. Canala, Prokoméo, 17 Dec 2006, one male. Pass on mining road from Poro to Kouaoua, one female (no date; Pauly et al. (2013b)).

##### Notes

This species has been observed in high altitude (up to 1350 m) maquis and forested areas on ultramafic substrates and high altitude (up to 850 m) forested areas on volcano sedimentary substrate.

#### Lasioglossum (Chilalictus) instabile

(Cockerell, 1914)*

4B12AA33-57F6-5A21-943B-4646E122743E

Halictus elliottii Rayment, 1929;
Lasioglossum instabilis (Cockerell, 1914)

##### Native status

Native

##### Distribution

Australia

Historical data in New Caledonia: Mt Koghis, 22 Dec 1992, one male and one female (Pauly et al. 2013b).

##### Notes

**Occurrence status**: data deficient.

#### Lasioglossum (Chilalictus) lanarium

(Smith, 1853)*

2BA27C4D-595E-5E17-8459-0291A2EC8F7E

Halictus mitchelli Cockerell, 1906;
Halictus lanuginosus Smith, F., 1879

##### Native status

Native

##### Distribution

Australia

Historical data in New Caledonia: Upper Vallée La Ni, 2 Nov 1992, two females (Pauly et al. 2013b).

##### Notes

**Occurrence status**: data deficient.

#### Lasioglossum (Chilalictus) polygoni

(Cockerell, 1929)

C5D5859C-1972-5592-A1AC-AE8A24BD52A0

##### Feeds on

*Polygonum* sp., *Agatealongipedicellata* (Pauly and Munzinger 2003), *Schinusterebinthifolius* (new records).

##### Native status

Native

##### Distribution

Australia

##### Notes

This species was first described from New Caledonia before being discovered in Australia. The status of the three subspecies should be confirmed through molecular analysis.

#### Lasioglossum (Chilalictus) polygoni austrocaledonicum

Pauly, Walker, Munzinger & Donovan, 2013

191FABCC-A410-5248-98D7-BCFE5BA9351F

##### Feeds on

Araliaceae: *Polysciasdioica* (native), *Polysciassessiliflora* (native), *Polyscias* sp. (native); Asteraceae: *Ageratumconyzoides* (alien); Cunoniaceae: *Pancheriasebertii* (native); Dilleniaceae: *Hibbertianana* (native); Elaeocarpaceae: *Elaeocarpusdognyensis* (native), *Elaeocarpusspeciosus* (native); Ericaceae: Stypheliacfcymbulae (endemic); Goodeniaceae: *Scaevolabeckii* (endemic); Mimosaceae: Leucaena leucocepala (alien); Myrtaceae: *Syzygiumquadrangulare* (native); Sapindaceae: *Cupaniopsisoedipoda* (endemic), *Cupaniopsis* sp. (native), *Guioavillosa* (native), *Litchichinensis* (alien) (Donovan et al. 2013); Araliaceae: *Polysciasbracteate*; Cunoniaceae: *Pancheriaternate*; Dilleniaceae: *Hibbertialucens*, *Hibbertiavirotii* (native); Ericaceae: *Dracophyllumramosum* (endemic); Mimosaceae: *Leucaenaleucocephala* (alien); Myrtaceae: *Syzygiumtetragonum*; Phellinaceae: *Phellinelucida* (endemic); Sapindaceae: *Ageratumconyzoides*, *Elaeocarpusdognyensis*, *Elaeocarpusspeciosus* (Pauly et al. 2013b).

##### Native status

Native

##### Distribution

Australia

Historical data in New Caledonia: Thi River Valley, 8 Nov 1940, one female. Mt Koghi, 28 Nov 1963, one female. Montagne des Sources, 29 Dec 1979, one female; 22 Oct 1980, one female; 25 Oct 1980, two females; 29 Jul 1981, one female. Mt Dzumac, 24 Feb 1980, one female; 4 Dec 2002, three females (including holotype). Thy Valley, 28 May 1980, two females; 18 Jun 1980, 3 females; 3 Jul 1980, six females; 8 Oct 1980, one female; 5 May 1981, 10 females. Thy Valley, Park entrance, 9 Oct 1980, two females. 2 km E Col de Mouirange, 10 Oct 1980, one female. Thy Valley Park, 12 Oct 1980, two females. Mt Koghis, 17 km NNE Nouméa, 24-26 Dec 1991, one female. Upper La Ni Valley, 2 Nov 1992, one female. Rivière Bleue Provincial Park, Trail to Upper Rivière Bleue, 5-16 Nov 1992, one female. Rivière Bleue, 16-17 Nov 1992, one female. Rivière Bleue Provincial Park, Rivière Bleue road, 20-28 Nov 1992, five females. Province Sud, Mt Ouin, 2 Dec 2000, one female. Mont Koghis, 4 Nov 2002, one female. Piste Ni-Dzumac, 7 May 2004, one male. Konguaoulou Nord, 27 Sep 2004, one female. Haute-Ni, 23 Oct 2004, two females; 25 Oct 2004, two females. Goro, embouchure de la Kuébuni, 14 Oct 2005, two females. Mont Koghis, 13 Jan 2007, one female (Pauly et al. 2013b). Dec 1979-Sep 2008, 25 females. Haute-Ni, 23 Oct 2004, one female (Donovan et al. 2013).

##### Notes

This species was observed in forested areas on ultramafic substrates including in high altitude (> 500 m).

#### Lasioglossum (Chilalictus) polygoni delobeli

Pauly & Munzinger, 2003

67CB3956-389B-5D39-8BAC-8283566D5F87

##### Feeds on

Verbenaceae: *Lantana* sp. (alien) (Pauly & Muzinger 2003); Araliaceae: *Polysciasdioica* (native); Arecaceae: *Cyphokentiacerifera* (endemic); Cunoniaceae: *Geissoisracemosa* (native); Dilleniaceae: *Hibbertialucens* (native); Ericaceae: *Dracophyllumramosum* (native); Melastomaceae: *Melastomamalabathricum* (native); Myrtaceae: *Myrtastrumrufo-punctatum* (endemic); Phellinaceae: *Phellinelucida* (native); Sapindaceae: *Litchichinensis* (alien) (Donovan et al. 2013); Sapindaceae: *Litchichinensis* (ailen) (Pauly et al. 2013b).

##### Native status

Endemic

##### Distribution

Historical data in New Caledonia: Col d’Amieu, 21 Jul 1977, three females (Pauly and Munzinger 2003, Donovan et al. 2013). Thy Valley, 3 Jul 1980, one female. Mt Aoupinie, 12 Dec 1980, one female. 6km NW Sarramea, 14-23 Nov 1992, four females. Rivière Bleue, 19-28 Nov 1992, one female. Col d’Amieu, 27 Nov 1992, one female. 9.3 km NW Sarraméa, 15 Jan 1996, one female. 9.1 km NW Sarraméa, 18-19 Jan 1996, one female. Mt Do, 15 Dec 2000, three females. Tchingou, 31 Mar 2001, one female. Koum River, 23 Nov 2001, one female. Haute Tchamba, 9 Nov 2002, one female. Col d’Amoss, 16 Nov 2002, two females. Towen, tribu de Tiendanite, 17 Nov 2002, two females. Mont Goroaté, 18 Nov 2002, three females. Pic du Grand Kaori, 21 Nov-29 Jan 2002, one female. Aoupinié, 25 Jul 2005, one female; Aoupinié, 15 Nov 2007, eight females; 11 Sep 2008, eight females. Vallée de la Tchamba, 26 Jul 2005, four females. Tchamba, 29 Oct 2005, one female. Hienghène Tao Mt Panié East Side, 9-26 Jan 2006, one male. Ponérihouen, forêt de l’Aoupinié, 12-13 Jan 2006, one female (Pauly et al. 2013b). Dec 1979-Sep 2008, one male and 29 females (Donovan et al. 2013).

##### Notes

This species has been observed in forested areas on both utramafic and volcano sedimentary substrates, with altitudes ranging from 5 to 1100 m.

#### Lasioglossum (Chilalictus) polygoni polygoni

(Cockerell, 1929)

8CF7938C-EEE4-50BB-BC62-F32F38756B3A

##### Feeds on

Asteraceae: *Blumealacera* (native); Elaeocarpaceae: *Elaeocarpusangustifolius* (native); Malvaceae: *Sidaacuta* (alien); Polygonaceae: *Antigononleptopus* (alien), Polygonum sp. (alien); Solanaceae: *Solanumtorvum* (alien); Violaceae: *Agatealongipedicellata* (endemic) (Donovan et al. 2013).

##### Native status

Native

##### Distribution

Australia

Historical data in New Caledonia: Bourail, 27 May 1927, one female; 1929, one female. Prony, 10 Feb 1999, one female (Pauly and Munzinger 2003). Dec 1979-Sep 2008, one male and seven females (Donovan et al. 2013). Oua Tom, 29 Apr 1981, 22 females. Koumac, 30 Apr 1981, one female. Prony, 10 Feb 1999. Magenta, 26 Apr 2001, one male. Païta, 28 May 2005, two females (Pauly et al. 2013b). Mont Dore: Rue des Trocas, 13 Apr 2022, two individuals; Saint Michel, 20 Apr 2022, one individual (Zakardjian et al. 2023b).

#### Lasioglossum (Chilalictus) tchambae

Pauly, Walker, Munzinger & Donovan, 2013

D35FCB57-E90E-522A-9CDC-3251AA16E47A

##### Feeds on

Melastomataceae: *Melastomamalabathricum* (native) (Donovan et al. 2013, Pauly et al. 2013b).

##### Native status

Endemic

##### Distribution

Historical data in New Caledonia: Dec 1979-Sep 2008, one male and one female (Donovan et al. 2013). Vallée de la Tchamba, 26 Jul 2005, one female (holotype). Aoupinié, (no date), two females (Pauly et al. 2013b).

##### Notes

This species has been observed in forested areas mostly in altitudes (> 500 m), on volcano-sedimentary substrates.

#### Lasioglossum (Chilalictus) webbi

Pauly, Walker, Munzinger & Donovan, 2013*

834093E3-78EB-5D64-AA75-9F4B567E7344

##### Native status

Endemic

##### Distribution

Historical data in New Caledonia: Ni Valley, 2 Nov 1992, one female (holotype). Mt Koghis, 22 Dec 1992, one male (paratype; Pauly et al. (2013b)).

##### Notes

**Occurrence status**: data deficient.

#### Lasioglossum (Parachilalictus) neocaledonicum

Pauly, Walker, Munzinger & Donovan, 2013

FB10E378-450C-54F4-9832-ACB51B6D3BEB

##### Feeds on

Ericaceae: Stypheliacfcymbulae (endemic) (Donovan et al. 2013, Pauly et al. 2013b).

##### Native status

Endemic

##### Distribution

Historical data in New Caledonia: Dec 1979-Sep 2008, one female (Donovan et al. 2013). Plateau de Dogny, 14 Nov 1992, one female. Mt Khogis, 25 Jan 1996, one female (paratype). Haute-Ni, 23 Oct 2004, one female (holotype; Pauly et al. (2013b)).

##### Notes

This species has been observed in forested areas on both volcano-sedimenatry and ultramafic substrates, mostly in altitudes 400-1000 m.

#### Lasioglossum (Parasphecodes) sulthicum

(Smith, 1853)*

4D3E988C-0F49-5AF1-B9E9-2C04E108F990

##### Native status

Native

##### Distribution

Australia

Historical data in New Caledonia: Mont Kogis, 30 Oct 1992, one female (Donovan et al. 2013, Pauly et al. 2013b).

##### Notes

**Occurrence status**: data deficient.

### Megachilidae

#### Lithurgus (Lithurgus) scabrosus

(Smith, 1859)

6755B673-BDCB-5D40-8E57-D5EE845F0EB4

Lithurgus albofimbriatus froggatti Cockerell, 1914;
Lithurgus albofimbriatus Sichel, 1867;
Lithurgus guamensis Cockerell, 1914;
Megachile scabrosus Smith, 1859

##### Feeds on

Convolvulaceae: *Ipomoea* sp. (native) (Cockerell 1929), *Ipomoeapescaprae* (native) (Pauly and Munzinger 2003); Convolvulaceae: *Ipomoea* sp. (Donovan et al. 2013).

##### Native status

Likely alien. Pauly & Villemant (2009) hypothesised that this species could have been accidentally introduced.

##### Distribution

Native range: Indonesia, Malaysia.

Alien range: Cook Islands, Fiji, French Polynesia, Guam, Hawaï, Micronesia, northern Mariana Islands, Papua New Guinea, Solomon Islands, Vanuatu.

Historical data in New Caledonia: Nouméa, Jul 1900. Dec 1979-Sep 2008, three males and four females (Donovan et al. 2013). Bourail, 26 May 1927, eight males and two females. Dge, Ile Uen, 6 Jun 1927, one female (Cockerell 1929). Koumac: Paagoumene, 12 Mar 1999, two males and one female (Pauly and Munzinger 2003). Ouvea, 9 Mar 2017, one male. Koumac, 2 May 2017, two females (Zakardjian et al. 2023b).

#### Megachile (Aethomegachile) laticeps

Smith, 1853

4A8D36B4-FBAE-5861-88F5-FB1520314C95

Megachile gadara Cameron, 1903;
Megachile mcgregori Cockerell, 1918;
Megachile metallescens Cockerell, 1918;
Megachile otriades Cameron, 1902;
Megachile penangensis Cockerell, 1918;
Megachile robbii Ashmead, 1904;
Megachile semperi Friese, 1905;
Megachile subignita Cockerell, 1918;
Megachile varidens Cameron, 1905;
Megachile caecina Cameron, 1903;
Megachile cinyras Cameron, 1902

##### Feeds on

*Durantarepens* (Pauly and Munzinger 2003); Convolvulaceae: *Ipomoeapes-caprae* (native); Myrtaceae: *Sannanthapinifolia* (native); Verbenaceae: *Durantaerecta* (alien) (Donovan et al. 2013); *Antigononleptopus*, *Cosmos* sp., *Cupheahyssopifolia*, *Durantaerecta*, *Ossinumbasilicum*, *Schinusterebinthifolius*, *Tridax* sp. (new records).

##### Native status

Likely alien. Pauly & Munzinger (2003) hypothesised that this species could be alien because it was captured in Noumea, while visiting an alien plant species.

##### Distribution

Native range: India to Malaysia, Nepal, Vietnam.

Alien rage: Singapore, Maldives, South Africa (remains to be confirmed), widely distributed in the Pacific Region (Vanuatu, Fiji, French Polynesia).

Historical data in New Caledonia: Nouméa, Jul 1957, one female. Dec 1979-Sep 2008, one male and one female (Donovan et al. 2013). Nouméa, 5 Feb 1999 one male and one female (Pauly and Munzinger 2003). Nouméa: Parc Zoologique et Forestier, 27 Feb 2019, one individual; 23 Mar 2019, one female; 04 Apr 2022, one individual; 22 Apr 2022, one individual; Centre culturel Tjibaou, 23 Mar 2019, two females; Rivière Salée, 14 Mar 2022, one individual; 16 Mar 2022, one individual; Petite Normandie, 29 Mar 2022, two individuals; 14 Apr 2022, one individual; Magenta Tours, 31 Mar 2022, four individuals; 12 Apr 2022, one individual; Tuband, 19 Apr 2022, one individual. Païta - La Tontouta: La Tontouta, 5 Apr 2019, one female. Mont Dore: La Conception, 23 Mar 2022, one individual; Rue André Burck, 1 Apr 2022, one individual; Vallon Dore, 20 Apr 2022, one individual (Zakardjian et al. 2023b).

#### Megachile (Callomegachile) rambutwan

Cheesman, 1936*

7388E0AF-FB5B-59FF-9A0A-C9792713EA05

Chalicodoma rambutwan (Cheesman, 1936)

##### Native status

Native

##### Distribution

Vanuatu

Historical data in New Caledonia: Nouméa, Aug 1900, one male (Donovan et al. 2013).

##### Notes

**Occurrence status**: data deficient.

#### Megachile (Callomegachile) umbripennis

Smith, 1853

F11A78C7-F708-56FF-A8C9-8F8794B47C81

Megachile domesticum Perkins, 1899;
Megachile lerma Cameron, 1908;
Megachile schauinslandi Alfken, 1898;
Megachile umbripennis var. atriventris Friese, 1903;
Chalicodoma umbripenne (Smith, 1853);
Megachile aureobasis Cockerell, 1919.

##### Feeds on

*Durantarepens* (Pauly and Munzinger 2003); Asteraceae: *Bidenspilosa* (alien); Polygonaceae: *Antigononleptotus* (alien); Protaceae: *Stenocarpus* sp. (native); *Cytisuscajan* (alien) (Donovan et al. 2013).

##### Native status

Likely alien. Pauly & Munzinger (2003) hypothesised that this species could be alien because it was captured in Noumea, while visiting an alien plant species.

##### Distribution

Native range: China, India, Malaysia, Myanmar, Nepal, Laos.

Alien range: United States, Singapore, Sri Lanka, Thailand, many Pacific islands including Cook Islands, Fiji, French Polynesia, northern Mariana Islands, Tonga, Hawaii (Ascher et al. 2016).

Historical data in New Caledonia: Dec. 1979-Sep 2008, eight males and two females. Noumea, 15 Oct 1980, two females (Donovan et al. 2013). Nouméa, 7 Feb 1999, two males (Pauly and Munzinger 2003). Koumac, 30 Apr 2017, two females. Mont Dore: La Conception, 23 Mar 2022, one individual (Zakardjian et al. 2023b).

#### Megachile (Eutricharaea) albomarginata

Smith, 1879

6780B3B2-3E97-5687-878D-1E57CA1365C9

##### Feeds on

Violaceae: *Agatealongipedicellata* (native), *Agatearufotomentosa* (native); Verbenaceae: *Durantaerecta* (alien) (Pauly and Munzinger 2003); Apocynaceae: *Parsonsiacrebriflora* (native), *Rauvolfiasemperflorens* (native), unknown (native); Araliaceae: *Tieghemopanax* sp. (native); Asteraceae: *Ageratum* sp. (alien), *Bidens* sp. (alien), *Cosmos* sp. (alien), *Tridaxprocumbens* (alien); Connaraceae: unknown (native); Dilleniaceae: *Hibbertiapodocarpifolia* (native), *Hibbertialucens* (native), *Hibberta* sp. (native); Goodeniaceae: *Scaevolabeckii* (native), *Scaevolaerosa* (native), *Scaevolamontana* (native); Lamiaceae: *Ocimumgratissimum* (alien); Mimosaceae: *Albizia* sp. (alien), *Leucaenaleucocephala* (alien), *Mimosadiplotricha* (alien); Myrtaceae: *Callistemon* sp. (alien), *Psidiumguajava* (alien), *Syzygiumlateriflorum* (native); Proteaceae: *Stenocarpus* sp. (native); Tiliaceae: *Triumfettarhomboidea* (alien); Verbenaceae: *Stachytarphetaindica* (alien) (Donovan et al. 2013); Apocynaceae: *Polygalapaniculata* (new records).

##### Native status

Native

##### Distribution

Fiji

Historical data in New Caledonia: Nouméa, 22 Jan-1 feb 1914, two females. Mt Mou, 12 Mar 1914, two females. Baie Ouemo, 28 Mar 1914, three males and seven females (Turner 1919). Houailou, 28 Oct 1925, two females (Cockerell 1939). Bourail, May 1927, two males and 14 females (Cockerell 1929). Dec 1979-Sep 2008, 14 males and 38 females (Donovan et al. 2013). Mont Koghi, 24 Feb 1986, one female. Rivière Bleue, 10 Jan 1999, four females; 11 Jan 1999, three females. Mandjelia, 27 Jan 1999, one female; 28 Jan 1999, one female. Nouméa, 7 Feb 1999, one male. Prony, 10 Feb 1999, one female and one male (Pauly and Munzinger 2003). Belep: Art Island 28 Feb 2017, two females. Païta - La Tontouta - La Tontouta, 22 Mar 2019, one female (Zakardjian et al. 2023b).

#### Megachile (Eutricharaea) australasiae

Dalla-Torre, 1896*

50F7712A-F021-53A0-AD56-956961681CEB

##### Native status

Native

##### Distribution

Its presence in Australia and Papua New Guinea remains to be confirmed.

Historical data in New Caledonia: Coinde, 12 Jan 1912, three males and one female. Bourail, 23 Jan 1912, three females (von Schulthess 1915).

##### Notes

The presence of this species is doubtful in New Caledonia as it was only mentioned by von Schulthess (1915) who spent one year on a field trip in New Caledonia between 1911 and 1912. As pointed out by Cockerell (1929), it is hard to understand how Sarasin and Roux could have collected this species and yet failed to find common endemic species (*Megachileaustralis* or *Megachilealbomarginata*). We then hypothesise that those individuals are misidentified individuals of *M.albomarginata*.

**Occurrence status**: data deficient.

#### Megachile (Eutricharaea) australis

Lucas, 1876

65D1393D-5E11-50F6-96AB-861609544A43

##### Feeds on

Aizoaceae: *Sesuviumportulacastrum* (native); *Wedeliatrilobata* (Pauly and Munzinger 2003); Amaranthaceae: *Achyranthesaspera* (alien); Apocynaceae: *Melodinus* sp. (native), *Rauvolfiasemperflorens* (native); Asteraceae: *Bidens* sp. (alien), *Cosmossulphureus* (alien), *Cosmos* sp. (alien), *Sphagneticolatrilobata* (alien), *Tridaxprocumbens* (alien); Convolvulaceae: *Ipomoeapes-caprae* (native); Dilleniaceae: *Tetracerabillardieri* (native); Euphorbiaceae: *Cleistanthusstipitatus* (native), *Euphorbia* sp. (alien); Goodeniaceae: *Scaevolasericea* (native); Labiatae: *Premnaserratifolia* (native); Mimosaceae: *Leucaenaleucocephala* (alien), *Mimosadiplotricha* (alien); Myrtaceae: *Melaleucaquinquenervia* (native), *Metrosiderosoperculata* (native), *Psidiumguajava* (alien), *Sannantha* sp. (native); Onagraceae: *Ludwigiaoctovalvis* (native); Poaceae: *Stenotaphrumsecundatum* (alien); Santalaceae: *Santalumaustrocaledonicum* (native); Surianaceae: *Surianamaritima* (native); Tiliaceae: *Triumfettarhomboidea* (alien); Verbenaceae: *Stachytarphetaaustralis* (alien);Zygophyllaceae: *Tribuluscistoides* (alien); *Desmodiumincanum* (native) (Donovan et al. 2013); *Poinsettia* sp. (Cockerell 1929), *Antigonumleptotus*, Orchidaceae, *Scaevola* sp. (new records).

##### Native status

Endemic

##### Distribution

Historical data in New Caledonia: Nouméa, Jun 1900, one female. Dec 1979-Sep 2008, 23 males and 32 females (Donovan et al. 2013). Nouméa, 23 Jan 1914, one male. Mt Canala, 12 Jun 1914, one female (Turner 1919). Bourail, May-Jun 1927, one male and three females. Mueo, May-Jun 1927, one male and four females. Tontouta, 28 May 1927, two males. Plum Farm, 4 Jun 1927, two males. Ile Nou, 10 Jun 1927, one female (Cockerell 1929). Plage du Mont Dore, 6 Feb 1999, one male, one female (Pauly and Munzinger 2003). Belep: Île Art, 2 Mar 2017, one female; 26 Apr 2017, one female; 27 Apr 2017, one female. Ouvea: 9 Mar 2017, one female. Tiga, 13 Apr 2017, one female. Koumac, 2 May 2017, two females. Nouméa: Petite Normandie, 31 Mar 2022, one individual; 12 Apr 2022, one individual (Zakardjian et al. 2023b).

#### Megachile (Eutricharaea) fullawayi

Cockerell, 1914*

07A203A9-9C4B-538E-9821-57C65A0E080B

##### Native status

Native

##### Distribution

Guam, Hawaï

Historical data in New Caledonia: Nouméa, Jul 1914, one male (Donovan et al. 2013).

##### Notes

**Occurrence status**: data deficient.

#### Megachile (Eutricharea) similis

Smith, 1879*

2171C6D1-1BFB-5B43-8B31-897140A8A7C4

Megachile zingowli Cheesman, 1936;
Megachile similis zingowli Cheesman, 1936

##### Native status

Native

##### Distribution

Vanuatu (Pauly and Villemant 2009).

Historical data in New Caledonia: Belep, Île Art, 2 Mar 2017, one male (Zakardjian et al. 2023b).

##### Notes

This species was previously considered as endemic to Vanuatu (Pauly and Villemant 2009). At this stage, it is difficult to know if it is native to New Caledonia and it has remained undetected since 2017 or if its presence is due to a recent human-induced displacement or a natural range expansion.

**Occurrence status**: data deficient.

#### Megachile (Hackeriapis) aurantiaca

Friese, 1905

A551F893-AAD4-54CE-A6DB-6A18E4DCB3A8

Chalicodoma aurantiaca (Friese, 1905);
Megachile quodi Vachal, 1907.

##### Feeds on

Violaceae: *Agatearufotomentosa* (endemic) (Pauly and Munzinger 2003); Apocynaceae: unknown (native); *Nephrodesmusferrugineus* (native); Flacourtiaceae: *Homaliumbetulifolium* (native); Liliaceae: *Dianella* sp. (native) (Donovan et al. 2013); *Acaciaspirorbis*, *Polygalapaniculata* (new records).

##### Native status

Endemic

##### Distribution

Historical data in New Caledonia: Dumbéa, 19 Dec 1979, one male. Dec 1979-Sep 2008, two males and six females (Donovan et al. 2013). Mandjelia, 27 Jan 1999, one male and two females; 28 Jan 1999, one female (Pauly and Munzinger 2003). Belep: Île Art, 28 Feb 2017, one male and two females; 1 Mar 2017, one female. (Zakardjian et al. 2023b).

#### Megachile ventralis

Smith, 1861 *

91568F3C-5317-512A-AAFB-538C81F5A6E3

##### Native status

Native

##### Distribution

Solomon Islands, Indonesia

Historical data in New Caledonia: Canala, 2 Jan 1912, one female (von Schulthess 1915).

##### Notes

**Occurrence status**: data deficient.

## Discussion

We found 23 publications, dated from 1897 to 2020, mentioning bee species in New Caledonia (Suppl. material 1). From the first published mention of New Caledonian bees to the present day, 51 bee species have been recorded, including 10 undetermined ones. Halictidae is the most represented family (26 species), followed by Megachilidae (11 species), Colletidae (10 species) and Apidae (4 species).

### Occurrence status

Based on their first and last year of capture, we applied occurrence status to each species. Thus, we can confirm the presence of 30 species in the archipelago and we lack data to do so for the remaining 21 species (i.e. data-deficient species).

Amongst the 21 data deficient species, 17 have been captured only once, three have been captured twice during the same year (i.e. *Lasioglossumwebbi* in 1992) and one is recorded for New Caledonia, based on personal communication with no specimen reported (i.e. Lasioglossum (Austrevylaeus) sp.). As bee samplings in New Caledonia prior to the 21^st^ century have been piecemeal and far from exhaustive, it is difficult to adjudicate on their current presence. Several hypotheses may explain why those species went unrecorded during recent samplings. First, several species may have been misidentified. For example, *Megachileaustralasiae* may be, in fact, a misidentification of *Megachilealbomarginata* (Cockerell 1929). Thus, this species should no longer be considered as present in New Caledonia. Then, some species may be cryptic and contemporary samplings may have not covered their period of activity and/or their New Caledonian range. Finally, we cannot exclude the possibility that some species may have become extinct. However, the lack of exhaustive samplings of the New Caledonian bee fauna does not allow us to discriminate between the two last hypotheses.

A significant part of the bee species of New Caledonia needs clarification (41%). An exhaustive sampling of the entire territory at different times of the year would provide a more precise picture of the New Caledonian bee fauna and potentially allow new species to be detected. A starting point to such a project could be to realise further samplings at periods and locations where data-deficient species were captured. This first step could provide additional information to support the presence or absence of these species. Moreover, it seems crucial to apply biomolecular analysis to the New Caledonian bee fauna. Bee species recorded from the archipelago have been described and identified exclusively, based on morphological criteria. This may have induced an over- or underestimation of the actual number of species (e.g. Praz et al. (2022)).

### Origin status

Amongst the 41 identified bee species recorded in New Caledonia, 20 were categorised as endemic, 12 as native and nine as alien. However, the status of endemic and native species may evolve over time. First, it is possible that some species considered as endemic to date actually have a wider distribution across the Pacific. For example, *Megachilealbomarginata* was considered endemic in Pauly and Munzinger (2003), but a later work showed that this species is also present in Fiji (Davies et al. 2013). Part of the southwest Pacific bee fauna remains unrecognised, as shown by the checklist of the bees of Vanuatu, in which five of 21 species are undetermined (Pauly and Villemant 2009). Thus, it would not be surprising if future studies reveal a wider distribution of certain species.

Concerning native bee species, biomolecular analysis could clarify the origin of certain species, notably megachilid bees. Biomolecular analysis suggested that Fijian megachilid bees are mostly, if not entirely, the result of anthropogenic displacements (Davies et al. 2013). Thus, the number of alien bee species in New Caledonia could be underestimated.

For now, alien bee species account for almost 20% of the New Caledonian bee fauna. Within the last seven years, three new alien bee species have entered the territory and established themselves (i.e. *Amegillapulchra* in 2016, *Braunsapispuangensis* in 2019 and *Hylaeusalbonitens* in 2022). New arrivals of alien bee species will likely continue in the years to come (Seebens et al. 2017) and their establishment raises serious concerns for native species. Alien bees may negatively impact native pollinators in a multitude of ways (i.e. competition for food and nesting resources, transmission of diseases and parasites, hybridation; Zakardjian et al. (2022)). Moreover, they may disrupt plant-pollinator interactions and impact native and alien plant reproductive success (Dohzono and Yokoyama 2010). While most native bees in New Caledonia are short-tongued species, almost all alien ones (except *Hylaeusalbonitens*) are long-tongued species. This morphological trait may allow alien bees to effectively pollinate alien plants that are poorly pollinated by native bees (i.e. awakening of sleeper weeds; Groom et al. (2014), Groom et al. (2017)). Additionally, if alien bees show morphological mismatches with native plant flowers, they may visit them without effectively transporting pollen, potentially disrupting their reproductive success.

### Conclusion

As in other Pacific islands (see Pauly and Villemant (2009) for Vanuatu and Naaz et al. (2021) for Fiji), the New Caledonian bee fauna is relatively poor, despite a remarkable and highly endemic plant diversity. Amongst the 51 species recorded through previous publications and recent samplings, only 30 are certainly or likely present. In order to have a more accurate picture of the New Caledonian bee fauna, there is a need to confirm species identifications and their original status through biomolecular analysis. Additionally, further samplings covering wider periods and areas are crucial to detect cryptic species and to potentially highlight local extinctions.

## Supplementary Material

XML Treatment for Amegilla (Zonamegilla) pulchra

XML Treatment for Apis mellifera mellifera

XML Treatment for Apis mellifera ligustica

XML Treatment for Braunsapis puangensis

XML Treatment for Ceratina (Neoceratina) dentipes

XML Treatment for Euhesma sp.*

XML Treatment for Euryglossina sp. 1*

XML Treatment for Euryglossina sp. 2*

XML Treatment for Hylaeus (Gnathoprosopis) albonitens

XML Treatment for Hylaeus sp. 1*

XML Treatment for Hylaeus sp. 2

XML Treatment for Hylaeus sp. 3*

XML Treatment for Leioproctus (Lamprocolletes) pacificus

XML Treatment for Leioproctus (Lamprocolletes) sp.*

XML Treatment for Palaeorhiza (Palaeorhiza) flavomellea

XML Treatment for Austronomia cheesmanae

XML Treatment for Austronomia doensis

XML Treatment for Austronomia loyali

XML Treatment for Austronomia neocaledonica

XML Treatment for Austronomia sicheli

XML Treatment for Homalictus aponi

XML Treatment for Homalictus cocos

XML Treatment for Homalictus curvistriae

XML Treatment for Homalictus heliotropiae

XML Treatment for Homalictus hienghenensis

XML Treatment for Homalictus koghiensis

XML Treatment for Homalictus mcphersoni

XML Treatment for Homalictus melanasiae

XML Treatment for Homalictus projectio

XML Treatment for Homalictus risbeci

XML Treatment for Homalictus risbeci crotalariae

XML Treatment for Homalictus sp. 1*

XML Treatment for Homalictus sp. 2*

XML Treatment for Lasioglossum (Austrevylaeus) sp.*

XML Treatment for Lasioglossum (Chilalictus) alticola

XML Treatment for Lasioglossum (Chilalictus) instabile

XML Treatment for Lasioglossum (Chilalictus) lanarium

XML Treatment for Lasioglossum (Chilalictus) polygoni

XML Treatment for Lasioglossum (Chilalictus) polygoni austrocaledonicum

XML Treatment for Lasioglossum (Chilalictus) polygoni delobeli

XML Treatment for Lasioglossum (Chilalictus) polygoni polygoni

XML Treatment for Lasioglossum (Chilalictus) tchambae

XML Treatment for Lasioglossum (Chilalictus) webbi

XML Treatment for Lasioglossum (Parachilalictus) neocaledonicum

XML Treatment for Lasioglossum (Parasphecodes) sulthicum

XML Treatment for Lithurgus (Lithurgus) scabrosus

XML Treatment for Megachile (Aethomegachile) laticeps

XML Treatment for Megachile (Callomegachile) rambutwan

XML Treatment for Megachile (Callomegachile) umbripennis

XML Treatment for Megachile (Eutricharaea) albomarginata

XML Treatment for Megachile (Eutricharaea) australasiae

XML Treatment for Megachile (Eutricharaea) australis

XML Treatment for Megachile (Eutricharaea) fullawayi

XML Treatment for Megachile (Eutricharea) similis

XML Treatment for Megachile (Hackeriapis) aurantiaca

XML Treatment for Megachile ventralis

37D6281A-8000-5926-A1AC-4633247091A6 10.3897/BDJ.11.e105291.suppl1 Supplementary material 1 Database of the bee species recorded for New Caledonia and references associated Data type Taxonomy, occurrences, origin status, references Brief description The sheet "references" compiles the publications reviewed to produce the checklist of the bees of New Caledonia. Each publication of interest is associated with an ID number.
The sheet "species" compiles the bee species and subspecies recorded for New Caleodnia, together with information on their taxonomy and occurrences ("first_occ" and "last_occ", respectively, refer to the first and last known date of capture of each species). The column "references" contains the ID numbers of the publications mentioning the according bee. File: oo_865372.xlsx https://binary.pensoft.net/file/865372 Zakardjian M, Jourdan H, Cochenille T, Mahé P, Geslin B

E8E992C0-B217-5D85-9F12-FEDF77F06C46 10.3897/BDJ.11.e105291.suppl2 Supplementary material 2 Map of the 14 sites sampled to produce the third dataset Data type Sampled sites Brief description Black squares represent the sampled sites, distributed from Nouméa (delimited by the bold black line) to the Col de Mouirange at the extreme East of the map. Ultramafic substrates appear in brown and non-ultramafic substrates in beige. File: oo_810705.PNG https://binary.pensoft.net/file/810705 Zakardjian M, Jourdan H, Cochenille T, Mahé P, Geslin B
