# Supplementary figures and images for: Checklist of the bees (Hymenoptera, Apoidea) of New Caledonia
Source: Biodivers Data J. 2023 Jul 31;11:e105291. doi: 10.3897/BDJ.11.e105291 (PMC10552698; doi:10.3897/BDJ.11.e105291)

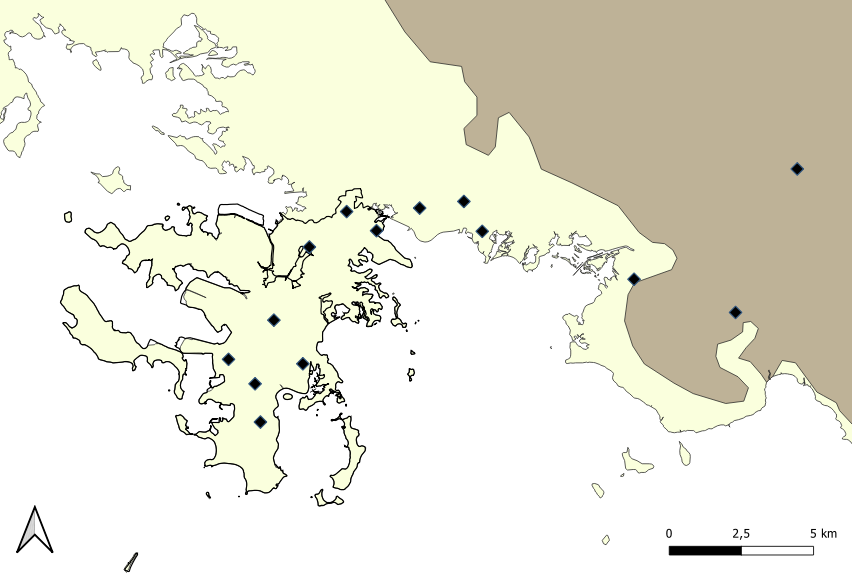

Supplement: Supplementary material 2 — Map of the 14 sites sampled to produce the third dataset [file bdj-11-e105291-s002.png]
